# Effectiveness of an implementation optimisation intervention aimed at increasing parent engagement in HENRY, a childhood obesity prevention programme - the Optimising Family Engagement in HENRY (OFTEN) trial: study protocol for a randomised controlled trial

**DOI:** 10.1186/s13063-016-1732-3

**Published:** 2017-01-24

**Authors:** Maria Bryant, Wendy Burton, Bonnie Cundill, Amanda J. Farrin, Jane Nixon, June Stevens, Kim Roberts, Robbie Foy, Harry Rutter, Suzanne Hartley, Sandy Tubeuf, Michelle Collinson, Julia Brown

**Affiliations:** 1 Leeds Institute of Clinical Trials Research, University of Leeds, Leeds, LS29JT UK; 2 Department of Nutrition, Gillings School of Public Health, University of North Carolina, Chapel Hill, NC 27599 USA; 3 Department of Epidemiology, Gillings School of Public Health, University of North Carolina, Chapel Hill, NC 27599 USA; 4 HENRY Head Office, 8 Elm Place, Old Witney Road, Eynsham, OX29 4BD UK; 5 Academic Unit of Primary Care, Institute of Health Sciences, University of Leeds, Leeds, LS2 9JT UK; 6 London School of Hygiene and Tropical Medicine, 15-17 Tavistock Place, London, WC1H 9SH UK; 7 Academic Unit of Health Economics, Leeds Institute of Health Sciences, University of Leeds, Leeds, LS2 9JT UK

**Keywords:** Engagement, Trial, Implementation, Childhood obesity, Parent, Recruitment

## Abstract

**Background:**

Family-based interventions to prevent childhood obesity depend upon parents’ taking action to improve diet and other lifestyle behaviours in their families. Programmes that attract and retain high numbers of parents provide an enhanced opportunity to improve public health and are also likely to be more cost-effective than those that do not. We have developed a theory-informed optimisation intervention to promote parent engagement within an existing childhood obesity prevention group programme, HENRY (Health Exercise Nutrition for the Really Young). Here, we describe a proposal to evaluate the effectiveness of this optimisation intervention in regard to the engagement of parents and cost-effectiveness.

**Methods/design:**

The Optimising Family Engagement in HENRY (OFTEN) trial is a cluster randomised controlled trial being conducted across 24 local authorities (approximately 144 children’s centres) which currently deliver HENRY programmes. The primary outcome will be parental enrolment and attendance at the HENRY programme, assessed using routinely collected process data. Cost-effectiveness will be presented in terms of primary outcomes using acceptability curves and through eliciting the willingness to pay for the optimisation from HENRY commissioners. Secondary outcomes include the longitudinal impact of the optimisation, parent-reported infant intake of fruits and vegetables (as a proxy to compliance) and other parent-reported family habits and lifestyle.

**Discussion:**

This innovative trial will provide evidence on the implementation of a theory-informed optimisation intervention to promote parent engagement in HENRY, a community-based childhood obesity prevention programme. The findings will be generalisable to other interventions delivered to parents in other community-based environments. This research meets the expressed needs of commissioners, children’s centres and parents to optimise the potential impact that HENRY has on obesity prevention. A subsequent cluster randomised controlled pilot trial is planned to determine the practicality of undertaking a definitive trial to robustly evaluate the effectiveness and cost-effectiveness of the optimised intervention on childhood obesity prevention.

**Trial registration:**

ClinicalTrials.gov identifier: NCT02675699. Registered on 4 February 2016.

## Background

Although the most recent data suggest a levelling off of the prevalence of childhood obesity in the United Kingdom, levels remain high and there are clear inequalities, with rates continuing to rise in more deprived and ethnic minority groups [1]. Childhood obesity impacts physiological and psychological health, which tracks into adulthood [2, 3], increasing the risk of morbidity and mortality [4, 5]. It imposes significant costs on the U.K. economy, with an expected sevenfold increase in related NHS costs by 2020 and a forecasted £2 billion annual spend by 2030 [6]. Tackling obesity is a national public health priority in terms of both treatment and prevention. Establishing healthy behaviours in early childhood is critical for optimum growth and development [7]. Further, poor eating patterns developed early can persist and are associated with chronic diseases in adulthood (e.g., cardiovascular disease, type 2 diabetes mellitus [8]). Once established, obesity is difficult to reverse [9], strengthening the case for primary prevention [10, 11]. To reverse trends in obesity, there is a clear need to engage parents in shaping healthy weight-related behaviours in their children.

Design, implementation and evaluation of interventions to prevent or treat obesity may be expensive and time-consuming, and researchers often report a lack of effectiveness [12–15]. Within the literature on childhood obesity, low parent enrolment and attendance at group-delivered programmes, along with a lack of reported behaviour change, are commonly described to have had a substantial impact on group dynamics, effectiveness and cost-effectiveness [16–18].

There is a need for pragmatic, ‘real-world’ evaluations of interventions to understand the generalisability of the interventions across everyday practice [19, 20]. Further, in order for interventions to be accurately implemented and interpreted, the underpinning behaviour change techniques need to be clearly defined [21]. Implementation optimisation strategies can then be adopted to target a specific intervention component [22]. It is imperative that parent engagement be improved across all parenting interventions delivered in community settings to optimise their impact and so that the degree to which they are effective can be assessed with confidence. In contrast to conventional pathways, in which implementation research is conducted following trials for clinical effectiveness [23], it is argued that there is a need for comprehensive early-phase (evidentiary) evaluation and enhancement of complex interventions such as those designed to prevent childhood obesity [24], prior to the conduct of large randomised controlled trials (RCTs). This novel approach ensures that factors which limit trial outcomes, such as low adherence, are minimised prior to dedicating the resources required to conduct a large trial which may identify no evidence of effectiveness, perhaps as a result of poor compliance.

The present trial involves the evaluation of an implementation enhancement ‘optimisation intervention’ of an existing preschool obesity prevention group programme, HENRY (Health Exercise Nutrition for the Really Young), to promote parent enrolment and attendance prior to establishing its clinical effectiveness and cost-effectiveness. HENRY is an 8-week group-delivered programme provided to parents of preschool children. It was developed in 2006 with joint funds from the Department of Health and the Department for Education (then called the *Department of Children, Schools and Families*) and is currently commissioned and delivered across the United Kingdom by 32 local authorities providing more than 150 programmes each year. It is delivered in community settings, often in children’s centres by children’s centre staff [25]. HENRY uses a responsive approach to provide practical guidance and improve parenting skills aimed at enhancing family lifestyle and children’s centre environments. Preliminary data indicate that HENRY may be effective at preventing childhood obesity and improving family health [25], although there is not yet evidence from an RCT.

Despite some indications of success of HENRY from audit [25] and qualitative data [26–28], process evaluation indicates implementation targets are often not met. Lack of involvement by some parents may be a threat to the viability of this programme and other, similar community-based interventions. The effectiveness of interventions in real-world conditions may not match the efficacy found in research studies [29]. This may be particularly relevant to population-based prevention programmes, in which parents may be less likely to be motivated to attend because their children show no clinical symptoms [30, 31]. Thus, it is imperative to create tailored methods to maximise parent participation and enhance successful implementation in childhood obesity prevention programmes.

Failure to attend interventions is complicated by issues of health inequalities; socio-demographic attributes; and, in some cases, challenges related to safeguarding [30, 32, 33]. Literature on non-attendance in other areas (clinical appointments) indicates that attendance is lower in certain populations and may be an indicator of vulnerability [34]. This is pertinent to obesity because prevalence rates continue to rise in children in lower socio-economic or ethnic minority groups. In addition to the anticipated public health benefits, the economic benefits of implementation optimisation in programmes such as HENRY are substantial, with one parenting intervention calculating an extra £800 cost per child in programmes that run with 8 parents compared with those running at the intended capacity of 12 parents [35].

Unlike the development of other robust behaviour change interventions, initiatives to improve parental attendance at clinical appointments have been confined largely to simple approaches such as text reminders [34, 36, 37]. To promote behaviour change, enhance the transparency of interventions and guide their generalisability, it is recognised that a systematic approach is used during intervention development, which is underpinned by theories of behaviour change and guided by an intervention-planning framework such as the Behaviour Change Wheel [24, 38]. Lessons can be learnt from child mental health research, in which many interventions have been developed and tested using robust theory-based approaches which carefully consider parent engagement [33]. Research in this field has explored perceived barriers to participation with suggested strategies to engage parents, including promotion of self-efficacy and treatment motivation. Such strategies perceive parents as the central agents of change and aim to modify the pre-treatment experience [33, 39]. RCTs evaluating parent engagement in mental health services [40] and drug misuse [41] indicate that parental engagement can be increased with a dedicated optimisation component, although a further trial focused on cultural relevance for child behaviour problems to optimise engagement [42] did not find any increase.

To our knowledge, there are no theory-based, multi-component optimisation interventions that have been developed to optimise parent engagement in obesity prevention programmes, and which have been evaluated using an RCT design. To meet this need, we developed a parent engagement optimisation intervention following Medical Research Council (MRC) guidance for development of complex interventions [38]. The Consolidated Framework for Implementation Research was used to understand contextual factors associated with the implementation of HENRY and their impact on parent engagement [43]. The Behaviour Change Wheel [44] provided guidance for the development of the intervention, which features use of the ‘COM-B’ model (capability, opportunity and motivation) to dissect behaviours into their individual and interacting components and consider which of these should be targeted in an intervention to bring about the desired behaviour change. A rapid ethnography was conducted in five children’s centres to understand the environmental, social, psycho-social, political and economic factors associated with parent engagement in HENRY. This study gathered data from approximately 190 h of observations, 22 interviews with children’s centre managers and staff, HENRY commissioners, and HENRY coordinators and facilitators. Six focus groups were also conducted with parents who had attended the programme. We convened an intervention development team consisting of experts in intervention development, obesity, applied health and behaviour change, a local authority representative, a HENRY parent champion, a parent who has attended the programme, and a representative from the HENRY team. The intervention team used evidence from the ethnography, from the literature, and from their own experiences and expertise to develop a multi-component optimisation intervention which attempts to promote parent engagement and positive behaviour change in families by supporting local authorities, children’s centres, HENRY staff, and parents using candidate implementation and delivery strategies (Career Development Fellowship CDF-2014-07-052). Here we report the protocol of our cluster randomised controlled trial (cRCT) (using routine process data) to evaluate the optimisation intervention for its ability to engage parents in HENRY prior to conducting a further evaluation of its clinical effectiveness.

## Methods/design

### Design

**Fig. 1:**
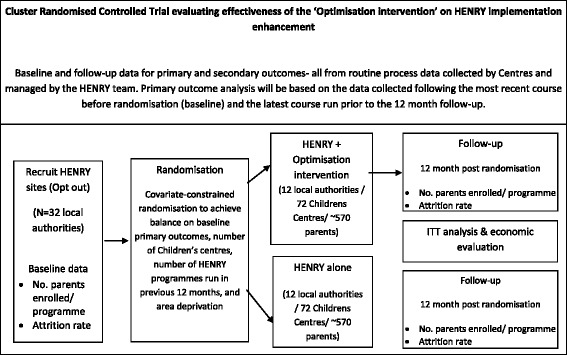
Trial design flow diagram. *HENRY* Health Exercise Nutrition for the Really Young, *ITT* Intention to treat

This study is a two-arm, multi-centre cRCT across 24 local authorities (local governments) (supporting approximately 144 children’s centres) in the United Kingdom to determine the effectiveness and cost-effectiveness of an optimisation intervention to promote parent engagement in the HENRY programme compared with standard HENRY practice (Fig. 1). Although the children’s centres deliver the HENRY programme, owing to the multi-component and interactive nature of the optimisation intervention, local authorities will be the units of randomisation (i.e., clusters) to prevent contamination between the randomised groups. Outcomes will be assessed through routinely collected data available for each HENRY programme at the parent and children’s centre levels. Information on commissioners’ willingness to pay (within local authorities) related to the optimisation intervention will be elicited. A process evaluation will be conducted alongside the cRCT in accordance with the MRC guidance on evaluating process in complex interventions [19].

### Objectives

Primary objectives of the trial are to determine the following:

1. The effect of the optimisation intervention applied to HENRY compared with standard HENRY in regard to increasing parent enrolment in HENRY programmes or reducing parent attrition within HENRY programmes
2. The willingness to pay of commissioners of the HENRY programme per additional level of parent engagement related to the optimisation intervention (Costs will be presented as an incremental cost per increase of parent engagement, so that commissioners are able to identify the point at which additional costs are acceptably offset by the benefits of engaging effectively with parents.)

Secondary objectives of the study are to determine:

1. The effect of the optimisation intervention on achieving combined parent enrolment, attrition and compliance targets
2. The effect of the optimisation intervention on parent compliance with HENRY programme content
3. The effect of the optimisation intervention on parent-reported family habits and lifestyle
4. The potential longitudinal impact (sustainability) of the optimisation intervention on enrolment and attrition in centres that provide data from more than one programme

### Setting

The study will be conducted in local authorities with children’s centres where staff are currently trained to deliver the HENRY programme. There are currently 32 local authorities (approximately 144 centres) in the United Kingdom running the programme, all of which provide process data to the central HENRY office for monitoring/quality assurance (QA) purposes. These data will be anonymised and transferred for analysis.

### Inclusion criteria

Local authorities comprising the following:

- Local authorities that commission HENRY and consent for their centres to be involved in the research
- Commissioning HENRY programmes delivered by trained staff who have been certified by HENRY

Children’s centres comprising the following:

- Centres providing routinely collected data for the most recent HENRY programme that they ran at the point of randomisation

### Exclusion criteria

Local authorities:

- Local authorities which plan to decommission the HENRY intervention during the period of the trial or which are not planning to run any HENRY programmes during the trial period

Children’s centres:

- Centres which were recruited in the development phase of the optimisation intervention (participating in the rapid ethnography)
- Centres which are not planning to run any HENRY programmes during the trial period

There will be no exclusions based on the demographics of children’s centres, but location will be monitored to ensure inclusion of those with diverse social and environmental characteristics. Randomisation will also stratify by area-level deprivation to ensure balance between trial arms.

### Recruitment and consent

**Fig. 2:**
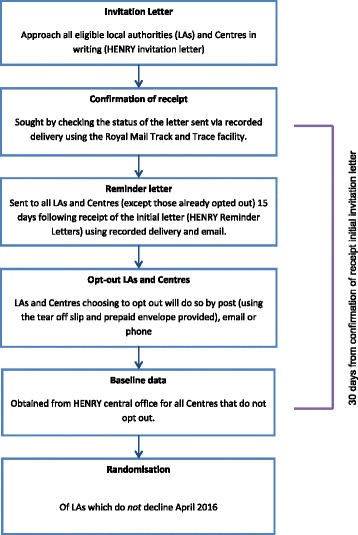
Recruitment/opt-out process. *HENRY* Health Exercise Nutrition for the Really Young, *LA* Local authorities

Local authorities and their centres across the United Kingdom will be identified from an existing database of HENRY delivery sites (see Fig. 2). Those meeting the eligibility criteria will be contacted by invitation letter (postal and electronic) issued jointly by the HENRY central office and the University of Leeds. HENRY is currently commissioned across the whole of the United Kingdom, with most sites situated within England.

Explicit consent will not be sought. Instead, opt-out consent will be sought at the cluster level (local authorities) and from the centres within each local authority. It will be possible for centres to decline participation in the randomisation, even if they are based within a consenting local authority. However, if a local authority declines to take part in the trial, none of its centres will be eligible to take part. Exclusion criteria related to further commissioning of HENRY (local authorities) and plans to run HENRY programmes (centres) will be self-reported by commissioners and centres, respectively. This opt-out method has been chosen because this is a low-risk trial in which anonymised data will be extracted remotely. It has been advocated to promote a simple and efficient trial design, and literature suggests that it is acceptable to participants [45]. It also replicates, as far as possible, the ‘real-life’ conditions under which such quality improvement initiatives are usually conducted in public health. The Leeds Institute of Clinical Trials Research (LICTR) has experience in using an opt-out approach and has found that it (1) is more likely than opt-in methods to provide a more representative or ‘typical’ participating sites; (2) appears more effective and efficient in promoting research participation by sites; and (3) is acceptable, provided that sufficient sensitivity and safeguards are applied [46]. In this instance, individual-/family-level consent is not required, because all patient-level data will be unlinked and anonymous and is routinely collected by centres and submitted to the central HENRY office. Centres will not be asked to conduct any additional data collection from parents for the purposes of this trial. Our parent advisory panel (patient and public involvement group) and trial steering committee (TSC) have both approved this process.

Local authorities and children’s centres will be given 30 days from the date on which the initial information letter is sent to decline participation (confirmed by recorded delivery). Centres that opt out after randomisation will be treated as ‘withdrawn’.

### Unit of randomisation

Local authorities will be randomised in a 1:1 allocation ratio (HENRY + optimisation intervention, HENRY as standard) by a statistician at LICTR, using an algorithm for covariate-constrained randomisation [47] to achieve a balanced allocation between the trial arms on (1) local authority baseline level of parental engagement with HENRY (proportion of centres recruiting a minimum of eight parents per programme, proportion of centres retaining at least 75% of parents for a minimum of five of eight sessions), (2) proportion of centres running at least one HENRY programme in 2015, (3) size of local authorities (number of children’s centres participating), and (4) area deprivation (proportion of centres in the least/most deprived quintiles as ranked by the 2015 Index of Multiple Deprivation at the Lower Layer Super Output Area) [48]. Randomisation will be performed for all local authorities at a single time point after baseline data are transferred, prior to the implementation of the intervention. All possible enumerations of allocation to the trial arms will be generated, and an imbalance statistic will be calculated for each allocation. A set of optimal allocations which minimise the imbalance will be provided, and, in accordance with the principles of International Conference on Harmonisation of Technical Requirements for Registration of Pharmaceuticals for Human Use guidance on randomness, the final allocation will be randomly selected from among this optimal set [49]. Results of the randomisation procedure will be sent by the LICTR statistician to the HENRY central office, which will be responsible for informing local authorities of their treatment allocation.

### Blinding

Owing to the nature of the intervention, it will not be possible to blind allocation within intervention sites or of those involved in the optimisation intervention training. Families attending the HENRY programme have been made aware that HENRY uses data anonymously for research, but they have not been told explicitly about the Optimising Family Engagement in HENRY (OFTEN) trial or whether their local authority has been assigned to the optimisation. HENRY is coordinated by a central team that is responsible for coordinating training and for providing support and QA to areas that commission HENRY. Because the HENRY central office is integral to the delivery of the optimisation intervention, it will not be possible to blind the HENRY central office (as it will communicate the randomisation allocation to all those responsible for its implementation); however, staff who are responsible for collating data and transferring it to LICTR will remain blinded to allocation. The chief executive officer of HENRY will be blinded to treatment allocation and will not attend TSC meetings. In addition, for the duration of the trial, the HENRY QA team have agreed to reallocated areas that they are responsible for, so that only one person will have contact with areas allocated to the optimisation intervention. Other QA staff will be aware that they have standard QA responsibility (e.g., answering questions, providing encouragement, checking on progress) for areas not randomised to the optimisation, but will not know the details or content of the optimisation intervention. To further avoid contamination, the details of the optimisation intervention will not be published until after the final analysis. Unplanned masking (unblinding) will be monitored and reported to the TSC.

### Intervention

#### HENRY

HENRY is an 8-week programme delivered in children’s centres with the aim of providing parents with skills, knowledge and confidence to support healthy lifestyles among their preschool children and their families, including parenting skills, emotional well-being and activity. The programme was set up in 2006 with the aim of reversing rising trends in school entry age obesity. HENRY is currently delivered within 32 local authorities across England and Wales by trained health and community practitioners who undergo two stages of training [26]. Stage 1, centre-level training, is a 2-day workshop designed to equip centre staff with knowledge and skills to promote and provide healthy nutrition within early years settings and support parents to provide healthy family lifestyles and nutrition for their families. The theoretical underpinning combines proven models of behaviour change, including the Family Partnership Model, motivational interviewing and solution-focused support. Stage 2, practitioner-level training to deliver the HENRY programme to families, is provided to approximately four practitioners per centre after completing Stage 1 to deliver the 8-week HENRY programme. The aim of this stage is to build parents’ skills, knowledge and confidence to change old habits, provide healthier nutrition for their young children, and encourage healthier lifestyles [25, 27]. Programme content includes sessions on lifestyle and eating habits (e.g., family meals), balancing healthy meals and snacks, child-appropriate portion sizes, parenting, physical activity and emotional well-being.

Current HENRY QA practice involves the review of process data by dedicated individuals in the HENRY central office with provision of written and oral feedback. This QA will continue in both trial arms and will be monitored. Staff delivering current QA activities will be blinded to treatment allocation.

#### HENRY + parent engagement optimisation intervention

We summarise only the key features of the optimisation intervention in the trial protocol to minimise contamination (i.e., uptake of initiatives of the intervention by local authorities randomised to the control arm) and maintain blinding. The optimisation intervention consists of interacting components delivered to local authorities, children’s centres and HENRY facilitators. It includes additional support to stakeholders, modification of HENRY marketing to clarify perceptions of the programme, and methods to modify the pre-programme experience for parents. Strategies have been developed to increase parent motivation to enrol in HENRY and promote parent self-efficacy to continue to attend. Implementation of the optimisation intervention will begin immediately following randomisation. All components of the optimisation intervention will be implemented within 6 months following randomisation.

#### HENRY as standard

Local authorities randomised to the control arm will continue to deliver HENRY programmes as per standard practice.

### Outcomes

#### Primary outcome

The effectiveness of the optimisation intervention will be determined by comparing parent engagement in HENRY + optimisation intervention vs HENRY alone. The co-primary outcomes are (1) the proportion of centres enrolling at least eight parents per programme and (2) the proportion of centres with at least 75% of parents attending five of eight sessions per programme. Both will be evaluated 12 months post-randomisation. The optimisation intervention will be deemed effective if either the enrolment or attrition goals are met.

#### Secondary outcomes

Secondary outcomes, measured 12 months post-randomisation (unless stated otherwise), include some further assessment of parent engagement to explore the degree to which the intervention was effective. Data from additional questionnaires which are routinely administered by HENRY facilitators at the beginning and end of each 8-week course will also been analysed to explore the potential impact of the optimisation on behavioural outcomes, outlined below:

- Proportion of centres achieving all targets for enrolment, attrition and parent compliance
- Parent adherence to HENRY programme content, defined as the proportion of parents reporting an increase of 0.5 in the daily frequency of consumption of fruits and vegetables by children per programme
- Impact of HENRY on parenting and family health, assessed by parent-report, including the following:
  - *Parenting self-efficacy* (assessed via four parenting confidence items with 5-point Likert scales, modified from the validated Parenting Self-Agency Measure [50]). This measure assesses the parents’ estimate of their ability to influence their child and environment to lead to positive development, which is a key goal of the HENRY approach. In order for the questionnaire to be feasible to administer as part of the service, HENRY chose to reduce the number of items to a single scale of parenting confidence. Internal consistency of these items in a previous HENRY evaluation was high, and internal consistency in this sample was high (Cronbach’s alpha = 0.82 at baseline, 0.74 at completion) [51].
  - *Eating behaviours* (based on the Golan Family Eating and Activity Habits Questionnaire [52]). This validated measure is sensitive to change and demonstrates high levels of reliability and validity. HENRY facilitators routinely administer six items related to family behaviours, included parent report of family sitting together for meals, watching television during mealtimes, consuming take-out meals, consumption of home-cooked foods, stopping eating when full and choosing healthy meals. Individual evaluation of these items will be undertaken because reliability for the scale was poor when previously tested using HENRY families (Cronbach’s alpha = 0.52 at baseline, 0.56 at completion).
  - *Family activity* (via a brief HENRY questionnaire asking parents to report the frequency that parents and children engage in exercise). This questionnaire has been developed bespoke to use within the HENRY evaluations and includes parental report of their own activities that result in breathlessness, with response categories ranging from none to <1 h, 2 h, 3 h or >3 h. Children’s activity is assessed using parental report as energetic play, with response categories ranging from none to 5–15 minutes, 20–30 minutes, 30 minutes to 1 h, or >1 h.
  - *Child screen time* (via a parent reported using the bespoke HENRY questionnaire). This assesses use of televisions, DVDs, computers, smartphones, and so forth with response categories ranging from none to <1 h, 1–2 h, 2–3 h, or >3 h.
  - *Intake of key indicator foods per day* (assessed via 14 items from a modified validated Food Frequency Questionnaire [53] completed in relation to the parent and child). Parents are asked to estimate how often (never, once per month, once per fortnight, 1–7 days per week) they consumed each of the 14 items or groups of foods (e.g., fresh fruit, sweets, chocolate, water), with space to report the number of times consumed. It has previously demonstrated sensitivity to change with parents attending HENRY sessions [25].
- Longitudinal impact of HENRY + optimisation intervention vs HENRY alone (parent engagement [enrolment and attrition] within centres during the 12-month follow-up in local authorities which provide data for more than one programme per centre at follow-up) (This evaluation will explore whether the optimisation intervention needs time to ‘bed-in’ and/or whether any early effects are maintained over time.)

### Process evaluation

A process evaluation will be conducted by an LICTR researcher in accordance with MRC guidance on evaluating process in complex interventions [19]. In brief, the aims of this evaluation will be to (1) examine the uptake, delivery and acceptability of the optimisation intervention across local authorities/children’s centres (reach); (2) explore the effects of individual intervention components at increasing parent engagement, along with potential unintended consequences; and (3) consider the utility of theories underpinning the intervention and approach used to develop the intervention design. Process measures will include quantitative measurement of centre uptake and fidelity of delivered trial components; qualitative and quantitative analysis of stakeholder perceptions of intervention components considering individual, organisational and contextual factors; quantitative measurement of enrolment methods used to recruit HENRY programme participants; and qualitative and/or quantitative measurement of predicted behaviour change outcomes among stakeholders targeted in the implementation optimisation. Quantitative data required for process evaluation will be gathered and reported on paper case report forms by a researcher within the LICTR, who will also collect qualitative data from a random selection of sites in person, including interviews and observations.

### Economic evaluation

Economic evaluation will model the benefits of optimisation intervention over the costs of implementation and will seek commissioners’ willingness to pay for this. It will include the costs required to deliver the intervention (gathered and transferred from HENRY) and the routinely collected outcome data.

The cost-effectiveness analysis in the present trial will include an array of trial endpoints (including parent enrolment and attrition rates) and costs. Cost-effectiveness acceptability curves will describe the probability that the optimisation intervention is cost-effective for a range of maximum monetary values that a decision-maker might be willing to pay for a particular unit change in outcome. Cost-effectiveness analysis leaves it to decision-makers to form their own view of the relative importance of these results [54]; therefore, in a second part of the work, we will conduct a meeting with commissioners to determine their willingness to pay per additional unit of effectiveness (parent engagement) before concluding whether the optimisation intervention can be considered cost-effective. We will use contingent valuation techniques and design an experiment to estimate the point at which commissioners consider that the costs of the programme are acceptably offset by the benefits of engaging effectively with parents.

### Sample size

We assumed that 25% of the 32 local authorities currently running HENRY will not be eligible or will opt out of the study, leaving 24 local authorities (12 per arm). Power calculations for this fixed sample size (with all eligible centres in the United Kingdom invited to take part) were therefore conducted to examine anticipated power for various intervention effects, at the 5% significance level, in each of the primary outcomes and for the composite endpoint (enrol at least eight parents per programme and retain ≥75% of parents attending five of eight sessions) (see Table 1 for scenarios). On the basis of data from previous HENRY programmes (all terms in 2014), we assumed an average of 6 children’s centres per local authority providing a total of 144 children’s centres (72 per arm), an intra-cluster correlation coefficient (ICC) between 0.05 and 0.1, a coefficient of variation in cluster size of 0.54, and the following estimates of the outcomes in the control sites (HENRY alone): 55% of centres will enrol at least eight parents per programme; 50% of centres will retain ≥75% of parents attending five of eight sessions; and 25% of centres will enrol at least eight parents per programme and retain ≥75% of parents attending five of eight sessions. Thus, with the anticipated number of centres (24 local authorities, 144 children’s centres), we will have at least 80% power to detect meaningful improvements in differences of between 25% and 30% in either of the primary endpoints or the composite endpoint at the 5% significance level, assuming the ICC is no greater than 0.05.

**Table 1:** Power calculations for enrolment and attrition endpoints for various estimates of the intervention effect and a fixed sample size of 144 children’s centres in 24 local authorities

Outcome in the control	Percentage point increase in intervention	Power for ICC = 0.1	Power for ICC = 0.05
Enrolment (≥8 parents per programme)			
55%	5%	6%	7%
	10%	15%	18%
	15%	29%	35%
	20%	49%	58%
	25%	70%	79%
	30%	87%	93%
Attrition (≥75% of parents attending five of eight sessions)			
50%	5%	6%	7%
	10%	15%	17%
	15%	28%	34%
	20%	47%	55%
	25%	67%	76%
	30%	84%	91%
Enrolment and attrition (at least eight parents per programme and 75% of parents attending five of eight sessions)			
25%	5%	7%	8%
	10%	17%	19%
	15%	31%	37%
	20%	49%	58%
	25%	67%	76%
	30%	82%	89%

*ICC* Intra-cluster correlation coefficient

### Data collection and transfer

In this trial, we will use routinely collected data for all primary and secondary outcomes, except for the process evaluation data, which will be gathered by an independent LICTR researcher. HENRY has designed a centralised process for evaluating programmes, developed for QA purposes and reporting to commissioners of the service. For the purpose of this trial, baseline data at the centre level include routine data on enrolment and attendance at programmes that have been run before randomisation. These data also include anonymous information on participant satisfaction, parent-reported compliance, parenting and family lifestyle (Table 2), which are gathered in questionnaires on the first and last days of each HENRY programme. This information is submitted to the central HENRY office (without any personal identifiers for the families), which check the data for completeness and collate the information in a.csv file.

**Table 2:** Data collection summary

Data	Screening	Randomisation	Baseline	On-going	Follow-up
Local authority level					
Cluster eligibility	X				
Stratification factors		X			
Programme enrolment			X		X
Programme attrition			X		X
Implementation data (e.g., fidelity)				X	
Parent level (anonymised routine)					
Infant diet (fruit and vegetable intake)			X		X
Family habits and lifestyle			X		X
Implementation/process data				X	
Intervention level					
Costs for intervention delivery for economic evaluation	X	X	X	X	X

X indicates when data are gathered

For the trial period, centre-level baseline data from the most recent programme delivered prior to randomisation (winter term 2015) will be used. Follow-up data will be gathered 12 months after randomisation. It will include routine data collected from the autumn and spring terms (2016–17), allowing 6 months for intervention delivery at all sites followed by the delivery of two terms of HENRY programmes. In accordance with the planned intention-to-treat (ITT) analysis, data will be collected from centres that are not compliant with the intervention. For those withdrawing from the trial, only data collected up to the point of withdrawal will be used.

All data transferred from HENRY to the LICTR will be in the form of unlinked, anonymised datasets and will be transferred via a secure, encrypted system. Only data for centres participating in the trial will be transferred. Data transfer agreements (including details on the sender, recipient, content of transfer/general purpose of transfer and any data-processing limitations) will be set up between HENRY and the LICTR prior to the transfer of any data. Queries pertaining to outliers or missing data will be sent to HENRY in accordance with LICTR standard operating procedures. Any new data that are identified following queries will also be transferred via the secure, encrypted system. Missing data, except individual data items collected via parent questionnaires, will be chased until it is received, confirmed as not available, or the trial is at analysis. For missing item-level data within questionnaires (item non-response), no attempts will be made to retrieve missing data; only missing questionnaires will be chased.

All data provided will be stored, handled and processed in accordance with the principles of the 1998 Data Protection Act, the operation of the agreement and the study publication policy. The rights for this data belong to the study sponsor, and no processing, including further data transfer in whole or in part to a third party, is permitted other than as stated in the data transfer agreements.

### Analysis

Statistical analysis of the quantitative elements of the trial is the responsibility of the LICTR statisticians. The analysis plan outlined in this section will be reviewed, and a detailed statistical analysis plan will be written and approved, before any formal analyses are undertaken. Statistical analyses will be carried out by the ITT principle, and statistical significance will be assessed at the two-sided 5% significance level. The ITT population is defined as analysis according to the randomisation and regardless of compliance with the protocol or withdrawal from the trial.

#### Primary analysis

The primary outcomes associated with enrolment or attrition will be compared between the intervention and control arms through a cluster-level analysis, using a weighted *t* test of cluster-level proportions and a two-stage process to adjust for covariates [55]. Intervention effects and corresponding 95% confidence intervals will be presented. As part of a sensitivity analysis, the reliability of random effects logistic regression will also be investigated.

In the primary analyses, missing data will be assumed to be missing completely at random; that is, analyses will be performed using complete cases (including those where sufficient item-level data exists). The extent of unit non-response (data missing from a whole course) will be assessed, and the reason for missingness and the missing data mechanism will be investigated.

A sensitivity analysis accounting for all participants in the ITT population assuming data missing at random, using multiple imputation, will be performed, and potential predictors of missingness will be investigated. The number of imputations will be determined using Bodner rule of thumb, where number of imputations is similar to the percentage of cases that are complete [56].

#### Secondary analysis

Adherence to HENRY programme content and the impact of the HENRY optimisation on parenting and family health will be analysed using a cluster-level analysis of either proportions or means, depending on the outcome, as described for the primary analysis. As part of sensitivity analyses, the degree of clustering at each level of the data structure, and the reliability of two-level (parents nested within centres or local authorities) and three-level (parents nested within centres within local authorities) random-effects models will also be investigated. The proportion of centres achieving combined enrolment, attrition and parent adherence will be analysed using the same methods described for the primary outcomes. The longitudinal impact of the intervention on parental engagement will be analysed among those local authorities providing data for more than one programme per centre at follow-up, using logistic regression with random effects adjusting for the time point of the programme.

Appropriate scoring manuals will be followed, and missing items within individual outcome measures will be treated according to instructions for that particular measure. For all other outcomes (without instructions for dealing with missing data), half rule will be used, substitution of the mean of the answered questions for that specific subscale for the missing responses as long as at least half the questions are answered [57]. If more than 50% of the items are missing, the outcome will be assigned as missing.

### Data monitoring

Trial supervision includes a core project team, a study management group, and a TSC. The core project team will comprise the chief investigator and a research assistant, with oversight from all other monitoring groups. The study management group will comprise the chief investigator, clinical trial research unit team (research assistant [WB], statisticians [BC and MC], data management, trial management [SH] and health economist [ST]). The TSC will comprise an independent chair (a professor of chronic disease and public health) plus independent expertise in statistics, qualitative and mixed-methods research, behaviour change and a parent (a volunteer from our parent advisory group). Because the trial is using routine data, a separate data monitoring and ethics committee was not convened. Rather, the independent TSC will adopt a safety monitoring role, and will establish a subcommittee to review safety issues should this become necessary. Serious adverse events are not anticipated, and, as such, a separate protocol for unmasking has not be produced. This will, however, be reviewed by the TSC as a regular item on the meeting agenda.

The TSC operates in line with the LICTR terms of reference as amended and agreed by TSC members at their first meeting. Data provided to the LICTR will be monitored for quality and completeness by the LICTR, using established verification, validation and checking processes. Clinical governance issues pertaining to all aspects of routine management will be brought to the attention of the TSC and, where applicable, individual local authorities.

### Trial organisation and administration

The trial is sponsored by the University of Leeds and coordinated by the LICTR (University of Leeds). The management group consists of the chief investigator and the study management group. Protocol amendments will be handled in accordance with LICTR standard operating procedures. Amendments required to the protocol and ethically approved documents will be identified by the trial researcher and the chief investigator. Amendments will be drafted and reviewed by members of the study management group and sponsor as appropriate. Substantial amendments to ethically approved documents will be submitted to the School of Medicine Research Committee for ethical opinion prior to implementation. A list of amendments will be included in the final report.

### Dissemination plan

A full dissemination plan will be written in accordance with those agreed by the funders. This includes dissemination to all stakeholders, including parents, and will be supported by our parent advisory group. The dissemination plan and publication plan will be developed by the study management group and agreed by the TSC. The policy will detail appropriate methods for determining writing groups, lead authors standard acknowledgement text to be included and necessary funding disclosure. The publication plan will detail potential paper titles (main findings and additional papers), authors (highlighting lead author) and time lines. Health Research Authority guidance for informing participants of the trial results will be applied [58].

## Discussion

Engaging parents to enrol and attend programmes to prevent obesity in their children’s early years is a challenge. This resounds with literature exploring the difficulties of recruiting parents to attend population-based programmes in the absence of health problems in their children [9, 30]. Early childhood provides an opportunity to intervene to establish healthy behaviours that are critical for optimum growth and development [7], but the failure to attract parents to programmes such as HENRY (particularly in populations theorised to have the greatest benefit) is a threat to the success and viability of such programmes. Our work has developed a theory-based intervention specifically targeting parent engagement with HENRY, with clear, transferable components for community-based interventions delivered to parents of young children. The early-phase evaluation of this optimisation intervention is described here, including methods to estimate the willingness of commissioners to pay for any additional costs borne by additional engagement activities. Ultimately, the true value of the optimised intervention will be ascertained following these discussions with the commissioners.

Implementation research is usually focused on the implementation of interventions of known effectiveness into routine practice and policy [23]. However, we intend to evaluate an approach to optimising the implementation of HENRY prior to a further evaluation of its clinical effectiveness. This approach is relatively novel and follows literature suggesting that complex intervention components should ideally be optimised and tested in evidentiary research [24]. Such an approach ensures that the future clinical effectiveness data relate to the impact that interventions can have once components have been enhanced. Similarly to other interventions (particularly prevention programmes), we recognised a need to increase parent engagement to enrol and attend HENRY programmes in order to improve the chances of a more successful, cost-effective definitive trial of clinical effectiveness. However, other components within complex interventions, such as content or delivery aspects, could warrant optimisation prior to effectiveness testing. A future pilot RCT is planned to test the feasibility of conducting a definitive trial of the effectiveness of HENRY to prevent childhood obesity (also within the National Institute for Health Research [NIHR] under Career Development Fellowship CDF-2014-07-052). This will occur regardless of the outcome of the present optimisation trial because there remains a need to establish an evidence base of an intervention that is already widely commissioned.

## Trial status

Recruitment and randomisation of local authorities is complete. Implementation is on-going and family enrolment (providing data on enrolment and attendance to the HENRY programme) was started in September 2016 and will continue until April 2017.

## Abbreviations

COM-B model: Capability, opportunity and motivation
cRCT: Cluster randomised controlled trial
HENRY: Health Exercise Nutrition for the Really Young
ICC: Intra-cluster correlation coefficient
ITT: Intention to treat
LA: Local authorities
LICTR: Leeds Institute of Clinical Trials Research
MRC: Medical Research Council
NIHR: National Institute for Health Research
OFTEN: Optimising Family Engagement in HENRY
QA: Quality assurance
RCT: Randomised controlled trial
TSC: Trial steering committee
